# Pratol, an *O*-Methylated Flavone, Induces Melanogenesis in B16F10 Melanoma Cells via p-p38 and p-JNK Upregulation

**DOI:** 10.3390/molecules22101704

**Published:** 2017-10-11

**Authors:** You Chul Chung, Seoyeon Kim, Jin Hwa Kim, Geun Soo Lee, Jung No Lee, Nam Ho Lee, Chang-Gu Hyun

**Affiliations:** 1 Department of Chemistry and Cosmetics, Jeju National University, Jeju 63243, Korea; jyc8385@hanmail.net (Y.C.C.); asksy613@naver.com (S.K.); namho@jejunu.ac.kr (N.H.L.); 2 Skin Science Research Institute, Itshanbul Cosmetics Co., Chungbuk 27651, Korea; kjhi@itshanbul.com (J.H.K.); aria@itshanbul.com (G.S.L.); 3 R&D Center, CoSeedBioPham Co., Chungbuk 28161, Korea; jnlee2000@hanmail.net

**Keywords:** pratol, B16F10 melanoma cell, flavone, melanogenesis, hypopigmentation, AKT, MAPK, p38, JNK

## Abstract

Tyrosinase is the rate-limiting enzyme critical for melanin synthesis. It controls pigmentation in the skin. Activation of tyrosinase is currently the most common approach in the development of tanning and haircare products. Pratol is a 7-hydroxy-4-methoxyflavone found in *Trifolium pratense*. In this study, we investigated the effects of pratol on melanogenesis. We also studied the mechanism of action of pratol in B16F10 mouse melanoma cells. The cells were treated with various concentrations (6.25, 12.5, 25, and 50 μM) of pratol to observe its effects. The results showed that pratol significantly increased melanin content and tyrosinase activity in the cells without being cytotoxic. In addition, pratol strongly increased the expression of tyrosinase and tyrosinase-related protein-1 and 2 by enhancing the expression of microphthalmia-associated transcription factor. Furthermore, pratol stimulated melanogenesis via the phosphorylation of p38, c-Jun N-terminal kinases (JNK), and extracellular signal–regulated kinase (ERK). The findings from an assay searching for the inhibitor revealed that SB203580 (a specific p38 inhibitor) or SP600125 (a p-JNK inhibitor) attenuated pratol-induced cellular tyrosinase activity whereas PD98059 (an ERK inhibitor) did not. Additionally, pratol interfered with the phosphorylation of p-AKT. We also found that pratol-induced melanogenesis was reversed by H89, which is a specific protein kinase A inhibitor. The results suggest that, owing to its multi-functional properties, pratol may be a potential tanning agent or a therapeutic agent for hair depigmentation in the cosmetic industry.

## 1. Introduction

Melanin is produced by melanosomes in melanocytes, which are located in the epidermal-dermal junction and deliver melanosomes to surrounding keratinocytes. Melanosomes are responsible for skin and hair coloration [1]. Melanin in the epidermis provides strong protection against photodamage caused by harmful UV radiation by scavenging free radicals or dispersing incoming UV light [2]. Hypopigmentation causes pigmentary disorders, such as vitiligo, albinism, and abnormal hair problems. Tyrosinase is the rate-limiting enzyme in melanogenesis. Therefore, activators of tyrosinase are used as tanning agents or in the treatment of hair depigmentation. They are also used as functional materials in the preparation of products for treating other medical disorders such as vitiligo and albinism [3,4,5].

Tyrosinase expression is regulated by microphthalmia-associated transcription factor (MITF). The major role of MITF is to increase tyrosinase expression by binding to M-box in the tyrosinase promoter. It also enhances the expression of other melanogenic enzymes such as tyrosinase-related protein (TRP)-1 and TRP-2 [6,7,8].

Recent investigations have indicated that mitogen-activated protein kinases (MAPKs), a family of serine/threonine kinases, including p38 MAPK, extracellular signal-regulated kinase (ERK), and c-Jun N-terminal kinase (JNK), which are particularly involved in regulating MITF expression [9,10,11]. In addition, it is reported that activation of protein kinase A (PKA) can induce MITF expression via phosphorylation of cyclic adenosine monophosphate (cAMP)-response element-binding protein (CREB). This increases the transcription of tyrosinase, which results in the activation of melanin synthesis [12,13,14]. Furthermore, recent studies have suggested that melanin synthesis is regulated by several signaling pathways, in particular, those involving ERK and phosphatidylinositol 3-kinase (PI3K)/AKT [15,16]. Recent studies have shown that some natural products upregulate melanogenesis in B16F10 melanoma cells through p-ERK and p-AKT downregulation [17,18]. For instance, cirsimaritin and *Ardisia crenata* extracts were found to increase melanogenesis via the downregulation of p-ERK or p-AKT expression. Therefore, regulation of the PI3K/AKT and MAPK signaling pathways has become a strategic target in the control of pigmentation. Moreover, several natural extracts and their ingredients have been reported to modulate the expression and activities of p-ERK and p-AKT. 

There have been several investigations on the stimulation of melanogenesis with methoxylated compounds such as sinensetin (5,6,7,3′,4′-pentamethoxyflavone), tangeretin (5,6,7,8,4′-pentamethoxy-flavone), nobiletin (5,6,7,8,3′,4′-hexamethoxyflavone), ferulic acid, and scoparone [19,20,21,22,23]. In the present study, we screened several compounds to discover new melanogenic activators in a preliminary investigation (Table 1).

We observed that, among the compounds studied, pratol (7-hydroxy-2-(4-methoxyphenyl)chromen-4-one), an *O*-methylated flavone in *Trifolium pratense* (red clover) strongly activated melanogenesis in B16F10 cells [24]. In this study, we therefore examined the effects of pratol on intracellular melanin amount, tyrosinase activity, and the signaling mechanism involved in tyrosinase expression in B16F10 melanocyte cells.

## 2. Results

### 2.1. Effects of Some Natural Compounds on Melanin Production and the Viability of B16F10 Melanoma Cells

**Figure 1:**
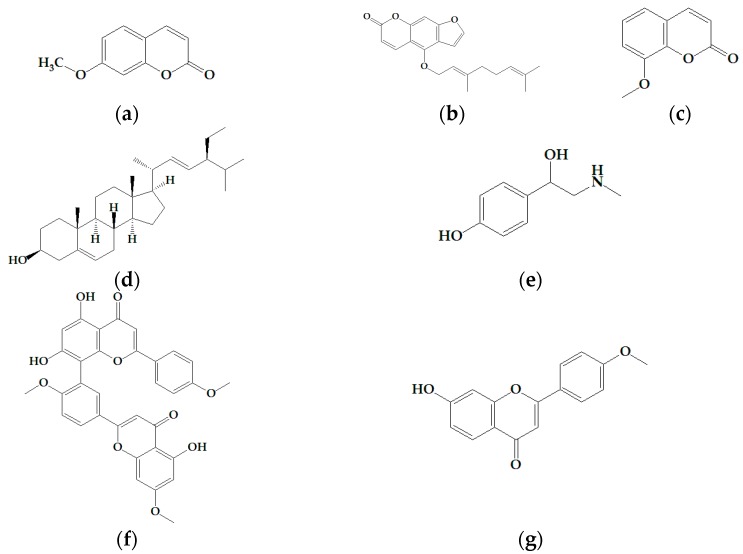
Structural formulae of (**a**) 7-methoxycoumarin; (**b**) bergamottin; (**c**) 8-methoxycoumarin, (**d**) stigmasterol; (**e**) synephrine; (**f**) sciadopitysin; and (**g**) pratol.

We tested the following natural compounds to find a potential candidate for a tanning agent or a therapeutic agent for hair depigmentation in the cosmetic industry: (**a**) 7-methoxycoumarin (7-methoxychromen-2-one); (**b**) bergamottin ((*E*)-4-[3,7-dimethyl-2,6-octadien-yl]oxy)-7*H*-furo[3,2-g][1]benzopyran-7-one); (**c**) 8-methoxycoumarin; (**d**) stigmasterol ((3*S*,8*S*,9*S*,10*R*,13*R*,14*S*,17*R*)-17-[(*E*,2*R*,5*S*)-5-ethyl-6-methyl-hept-3-en-2-yl]-10,13-dimethyl-2,3,4,7,8,9,11,12,14,15,16,17-dodecahydro-1*H*-cyclopenta[*a*]phenanth-ren-3-ol); (**e**) synephrine (4-[1-hydroxy-2-(methylamino)ethyl]-phenol); (**f**) sciadopitysin (Amentoflavone-7,4′,4′′′-trimethyl ether), and (**g**) pratol (Figure 1).

As shown in Table 1, pratol strongly upregulated melanin production in a dose-dependent manner. Therefore, additional experiments were focused on investigating whether pratol stimulates melanogenesis and melanogenic protein expression in mouse B16F10 melanoma cells.

### 2.2. Effects of Pratol on Melanin Production and the Viability of B16F10 Cells

**Figure 2:**
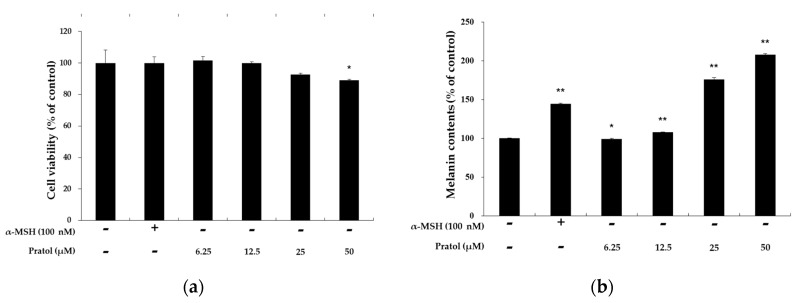
Effects of pratol on melanin production and the viability of B16F10 melanoma cells. The cells were treated with pratol (6.25, 12.5, 25, or 50 μM) for 48 h. α-MSH (100 nM) was used as the positive control. (**a**) Cell viability and (**b**) melanin content are expressed as percentages compared to the respective values obtained for the control cells. The data are presented as mean ± standard deviation (SD) of at least three independent experiments. * indicates *p* < 0.05, whereas ** indicates *p* < 0.01.

B16F10 cells were treated with pratol (6.25, 12.5, 25, or 50 μM) for 48 h to determine the maximum concentration applied to the future experiment. α-Melanocyte-stimulating hormone (MSH, 100 nM) and Dulbecco’s modified Eagle’s medium (DMEM, solvent) were used as the control agents. As shown in Figure 2a, there was no significant difference in the proliferation rate between control and pratol-treated cells. These results indicate that the effect of pratol on melanin biosynthesis is not attributable to cell proliferation at the abovementioned concentrations.

To assess the melanogenic activity of pratol, we measured melanin content in α-MSH-untreated B16F10 melanoma cells after exposing to pratol for 48 h. As shown in Figure 2b, pratol significantly increased cellular melanin content in a dose-dependent manner.

### 2.3. Effect of Pratol on Tyrosinase Activity in B16F10 Cells

**Figure 3:**
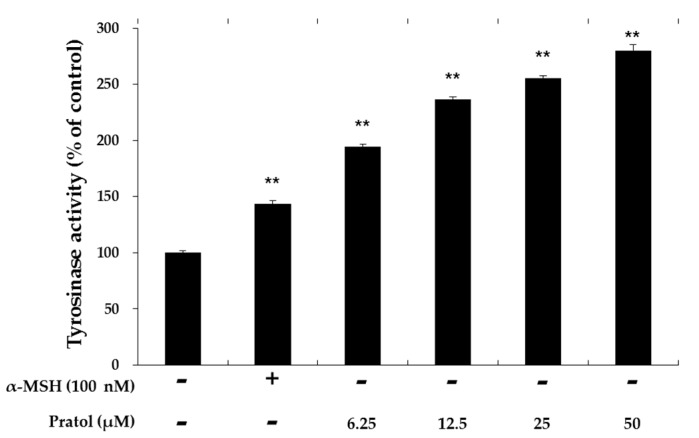
Effect of pratol on tyrosinase activity. B16F10 cells were treated with pratol (6.25, 12.5, 25, or 50 μM) for 72 h. α-MSH (100 nM) used as the positive control. The results are expressed as percentages compared to the value obtained for the control cells. The data are presented as mean ± SD of at least three independent experiments. ** indicates *p* < 0.01.

Since tyrosinase is a crucial enzyme in the regulation of melanogenesis, we examined the effect of pratol on the up-regulation of tyrosinase activity in B16F10 cells. As shown in Figure 3, tyrosinase activity was upregulated by pratol in a dose-dependent manner. This suggests that pratol increases melanin synthesis through tyrosinase upregulation in B16F10 cells. 

### 2.4. Effect of Pratol on the Protein Expression of Melanogenic Enzymes and MITF

Tyrosinase, TRP-1, and TRP-2 play important roles in melanogenesis [25]. MITF is a well-known regulator to affect the activity of the abovementioned enzymes. It binds to M-box in the promoters of tyrosinase and melanogenic enzymes, thereby inducing their expression. In order to determine the mechanism by which pratol stimulates melanin biosynthesis, we investigated the expression of MITF and its target genes, including tyrosinase, TRP-1 and TRP-2, by western blotting analyses.

**Figure 4:**
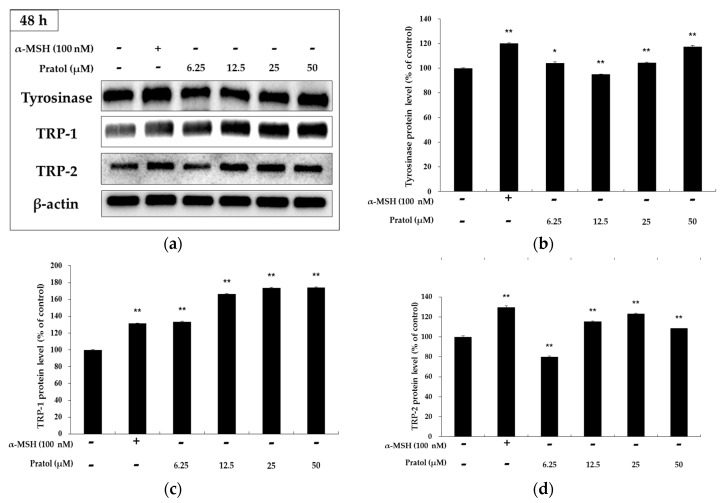
Effect of pratol on TRP-1, TRP-2, and tyrosinase expression in B16F10 cells. Cells were treated with various concentrations of pratol (6.25, 12.5, 25, or 50 μM) for 48 h. Protein levels were examined by western blotting. (**a**) Result of western bolting, and protein level of (**b**) tyrosinase; (**c**) TRP-1 and (**d**) TRP-2. Results are expressed as a percentage of the control. The data are presented as mean ± SD of at least three independent experiments. * indicates *p* < 0.05, whereas ** indicates *p* < 0.01.

**Figure 5:**
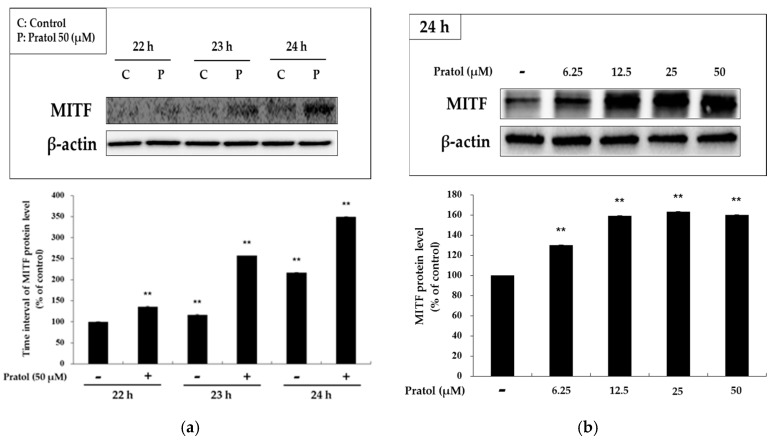
Effect of pratol on MITF expression in B16F10 cells. (**a**) Cells were treated with pratol (50 μM) at different time intervals; (**b**) Cells were treated with various concentrations of pratol (6.25, 12.5, 25, or 50 μM) for 24 h. Protein levels were examined by western blotting. Results are expressed as a percentage of the control. The data are presented as mean ± SD of at least three independent experiments. ** indicates *p* < 0.01.

As shown in Figure 4, B16F10 murine melanoma cells treated with pratol showed a significantly increased expression of tyrosinase, TRP-1, and TRP-2. To understand the transcriptional regulation of tyrosinase and *TRP-2* genes, we investigated the effect of pratol on the expression of MITF. As shown in Figure 5, results of the analysis showed time- and concentration-dependent increase in the expression of MITF and the melanogenic proteins following treatment of B16F10 cells with pratol.

### 2.5. Pratol Suppresses p-AKT Expression in B16F10 Melanoma Cells

**Figure 6:**
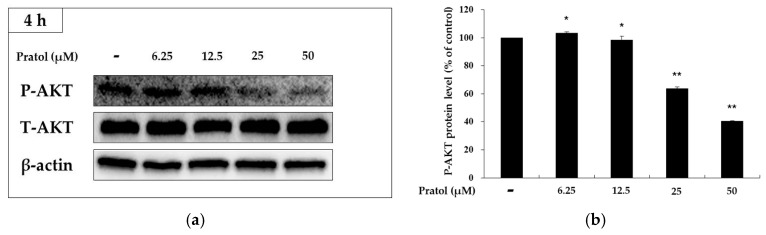
Effects of pratol on AKT phosphorylation. B16F10 cells were treated with various concentrations of pratol (6.25, 12.5, 25, or 50 μM) for 4 h. (**a**) Protein expression levels were investigated by western blotting; (**b**) Results are expressed as a percentage of the control. The data are presented as mean ± SD of at least three independent experiments. * indicates *p* < 0.05, whereas ** indicates *p* < 0.01. P: Phosphorylated, T: Total.

Previous studies have revealed that AKT signaling pathways participate in regulating melanogenesis [17,18]. Therefore, upstream signaling pathways underlying the hypopigmentary effect of pratol were investigated. The expression of AKT and its phosphorylated form were analyzed by western blotting. The results clearly showed that pratol reduced AKT phosphorylation in a dose-dependent manner. Pratol significantly reduced AKT phosphorylation at a concentration of 50 μM (Figure 6). These findings show that pratol strongly enhances melanogenesis by markedly reducing the phosphorylation of AKT.

### 2.6. Pratol Increases p-ERK, p-p38, and p-JNK Expression in the MAPK Pathway

**Figure 7:**
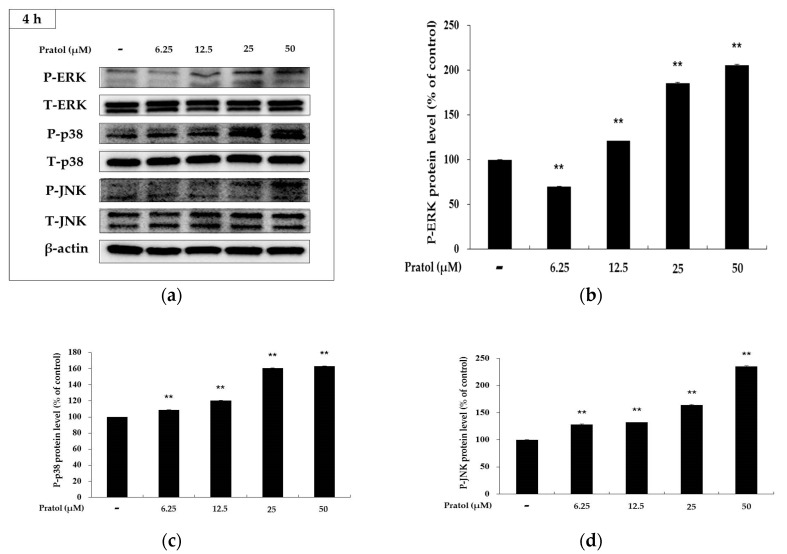
Effects of pratol on the phosphorylation of p-ERK, p-p38, and p-JNK. B16F10 cells were treated with pratol at the indicated concentrations for 4 h. (**a**) Result of western bolting, and protein level of (**b**) p-ERK, (**c**) p-p38 and (**d**) p-JNK. The data are presented as mean ± SD of at least three independent experiments. ** indicates *p* < 0.01. P: Phosphorylated, T: Total.

**Figure 8:**
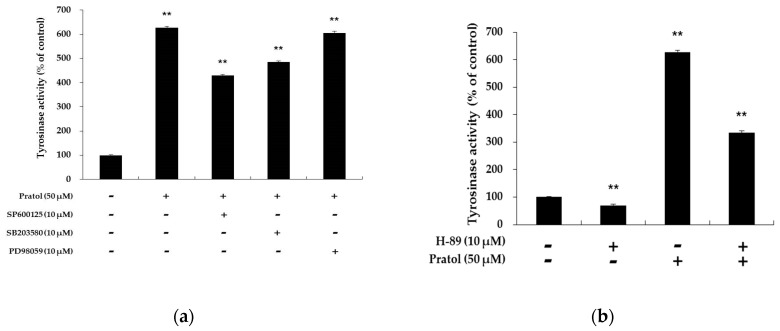
Effect of MAPK inhibitors on pratol-induced tyrosinase activation in B16F10 cells. To determine the involvement of MAPK enzymes in melanogenesis, a cellular tyrosinase activity assay was conducted using the following MAPK inhibitors: (**a**) SP600125 (JNK inhibitor), SB203580 (p38 inhibitor), and PD98059 (ERK inhibitor). To observe the involvement of the cAMP pathway in melanogenesis; (**b**) a PKA inhibitor (H-89) was subjected to a cellular tyrosinase activity assay. The data are presented as mean ± SD of at least three independent experiments. ** indicates *p* < 0.01.

Recently, the MAPK signaling pathway has been reported to be associated with melanogenesis. The activation of p38 MAPK, ERK, and JNK in B16F10 cells was examined to elucidate the mechanism underlying the hypopigmentary effect of pratol. As shown in Figure 7, pratol significantly increased phosphorylation of p38 MAPK, ERK, and JNK in a dose-dependent manner. Given that the MAPKs were activated in B16F10 cells, whether they were involved in the up-regulation of tyrosinase activity was then assessed. We treated the cells with 10 μM of SB203580, SP600125, and PD98059, which are inhibitors of p38, JNK, and ERK, respectively. As shown in Figure 8a, SB203580 or SP600125 markedly attenuated pratol-induced improvement of tyrosinase activation in B16F10 cells. This indicated that pratol-induced tyrosinase up-regulation was correlated with p38 and JNK activation.

### 2.7. Pratol Induces Melanogenesis through the PKA-Dependent Signaling Pathway

PKA activation and subsequent CREB phosphorylation are major stimulatory signals for MITF transcription. To further substantiate the involvement of CREB in pratol-induced melanogenesis, B16 melanoma cells were treated with the H-89, a PKA inhibitor in the presence or absence of pratol treatment. As shown in Figure 8b, pratol in combination with H-89 significantly decreased tyrosinase activity more than only patrol did. Therefore, pratol directly enhanced tyrosinase activity and increased the expression of melanogenic genes through a PKA-dependent signaling pathway.

## 3. Discussion

It is reported that the 4′-*O*-methyl group on the B-ring of flavonoids plays a crucial role in the stimulation of melanogenesis in B16F10 melanoma cells. Nevertheless, the pathways underlying the induction of melanogenesis by 4′-*O*-methylated flavonoids are not fully understood. In this study, we evaluated the effect of pratol, a 7-hydroxy-4-methoxyflavone, on melanogenesis in B16F10 mouse melanoma cells. We also used this mechanistic study to understand the signaling pathways. Our results show that pratol induces melanogenesis in B16F10 melanoma cells. We also investigated the effect of pratol on melanin synthesis by evaluating melanin content and tyrosinase activity in the cells. In addition, the expression of melanogenic enzymes (tyrosinase, TRP-1, and TRP-2), as well as phosphorylation of their regulators (MITF, AKT, MAPKs), in the cells were investigated. As expected, our results showed that pratol dose-dependently induced melanogenesis in the cells. The results of a 3-(4,5-dimethylthiazol-2-yl)-2,5-diphenyltetrazolium bromide (MTT) assay showed that pratol decreased cell proliferation rate at concentrations above 100 μM; however, it did not affect cell viability at all tested concentrations (6.25–50 μM). In addition, we found that pratol-induced melanogenesis was accompanied by increased expression of tyrosinase, TRP-1, and TRP-2. 

The abovementioned three enzymes are transcriptionally regulated by MITF, a transcription factor that plays a pivotal role in melanin synthesis. Thus, blocking or activating signaling pathways involved in the modulation of melanogenic enzymes may be beneficial in the development of skin whitening and/or tanning agents. In order to evaluate whether pratol affects the MITF regulator, we conducted a western blot analysis. Our results showed that pratol-induced increase in MITF expression reached a maximum level after 24 h of treatment. Furthermore, the results showed that pratol (6.25, 12.5, 25, or 50 μM) increased MITF expression in a dose-dependent manner (Figure 5b). These results indicate that pratol exerts a stimulatory effect on pigmentation by increasing the expression of *MITF* gene.

The cAMP pathway plays a crucial role in melanogenesis. In a previous study, cAMP-elevating agents inhibited PI3K and AKT phosphorylation, resulting in the binding of MITF to the tyrosinase promoter, which resulted in the stimulation of melanogenesis [14,26]. To evaluate whether pratol affects this signaling pathway, we assessed the phosphorylation status of AKT by western blot analysis. As shown in Figure 6, pratol significantly inhibited the phosphorylation of AKT. The results also show that pratol induces melanogenesis through p-AKT inhibition (Figure 8b).

In the MAPK pathway, p38 and JNK phosphorylation are implicated with the melanogenesis by enhancing MITF expression [27,28,29]. In contrast, activation of ERKMAP kinases diminishes the tyrosinase activity and melanin production in B16F10 cells [30]. In the present study, the western blot assay showed that pratol increased the expression levels of not only p-p38 and p-JNK but also p-ERK (Figure 7). Therefore, we examined tyrosinase activity using SB203580, SP600125, and PD98059 in order to identify which enzyme pathway is involved in melanogenesis [18,31]. We found that, compared to treatment with pratol alone, co-treatment with PD98059 and pratol did not change tyrosinase activity significantly (Figure 8a). This indicated that the ERK pathway does not affect melanogenesis.

The data obtained from this investigation show that pratol induces melanogenesis in B16F10 melanoma cells without causing cytotoxicity. Furthermore, we found that pratol enhanced p-p38 and p-JNK expression in the MAPK pathway. It also activated cAMP production, which upregulated MITF expression and finally increased melanogenesis. These findings indicate that pratol may be useful in treating hypopigmentation disorders. Additionally, it could be used in the formulation of skin-tanning and anti-white hair products.

## 4. Materials and Methods

### 4.1. Chemicals and Reagents

Pratol was purchased from Extrasynthese (Genay CEDEX, France). DMEM, fetal bovine serum (FBS), penicillin/streptomycin, trypsin-ethylenediaminetetraacetic acid, and PD98059 were purchased from Thermo Fisher Scientific (Waltham, MA, USA). Dimethyl sulfoxide (DMSO), α-MSH, NaOH, MTT, radioimmunoprecipitation assay (RIPA) buffer, and H-89 were obtained from Sigma-Aldrich (St. Louis, MO, USA). Antibodies against tyrosinase, TRP-1, TRP-2, and MITF were purchased from Santa Cruz Biotechnology (Dallas, TX, USA). p-p38, p38, p-JNK, JNK, p-ERK, ERK, p-AKT, AKT, and β-actin antibodies were obtained from Cell Signaling Technology (Danvers, MA, USA). SP600125 and SB203580 were purchased from Cayman Chemical (Ann Arbor, MI, USA) and Calbiochem (San Diego, CA, USA), respectively. Enhanced chemiluminescence (ECL) kit and 2× Laemmli sample buffer were obtained from Biosesang (Sungnam, Gyeonggi-do, Korea) and Bio-Rad (Hercules, CA, USA), respectively.

### 4.2. Cell Culture

B16F10 mouse melanoma cells were cultured in phenol-red-free DMEM with 1% penicillin and 10% FBS at 37 °C in a humidified atmosphere containing 5% CO_2_. 

### 4.3. Cell Viability Assay

Cell viability was determined by MTT assay. Briefly, B16F10 melanoma cells were incubated for 24 h in the culture medium. The cells (2.0 × 10^4^ cells/well) were seeded into 24-well plates and incubated with various concentrations of pratol (6.25, 12.5, 25, or 50 μM) for 48 h. After incubation, the cells were treated with MTT (dissolved in DMEM to 0.5 g/L) for 3 h. The solution removed after treating with MTT, and DMSO was added to dissolve the formazan crystals. Absorbance was measured at 550 nm on a spectrophotometric microplate reader (Tecan, Mannedorf, Switzerland).

### 4.4. Measurement of Melanin Content

Melanin content in the cells was measured using a previously described method with slight modifications [32]. Briefly, B16F10 cells were incubated for 24 h in the culture medium. The cells (5.0 × 10^4^ cells/well) were seeded into 6-well plates and incubated for 48 h with various concentrations of pratol (6.25, 12.5, 25, or 50 μM) for 48 h. α-MSH (100 nM) was used as the positive control treatment. The cell pellets were harvested and dissolved in 1 mL of 1 N NaOH containing 10% DMSO at 70 °C for 1 h. Next, 200-μL aliquots of the media were placed in 96-well plates and absorbance was measured at 405 nm using an ELISA reader (Tecan, Mannedorf, Switzerland). Protein concentration was quantified by bicinchoninic acid (BCA) protein assay.

### 4.5. Intracellular Tyrosinase Activity

B16F10 cells were incubated for 48 h in the culture medium. The cells (1.0 × 10^5^ cells/well) were seeded into 100-mm dishes for 48 h and then incubated with various concentrations of pratol (6.25, 12.5, 25, or 50 μM) and α-MSH (100 nM) for 72 h. After incubation, the cells were collected in a conical tube for centrifugation at 11,000× *g* for 3 min. Next, supernatants were removed, and cells were lysed with RIPA buffer and protease inhibitor cocktail (1.0%). Cell lysates were vortexed every 10 min for 1 h and then centrifuged at 13,000× *g* for 10 min. The quantity of each cell lysate was adjusted using 0.1 M sodium phosphate buffer (pH 6.8) to ensure equal protein concentrations. Next, 20 μL of each lysate and 80 μL of L-dopa (2 mg/mL) were mixed in the wells of a 96-well plate. After incubation at 37 °C for 2 h, absorbance was measured at 490 nm by ELISA.

### 4.6. Western Blot Assay

B16F10 cells (1.0 × 10^5^ cells/well) were seeded into 100-mm dishes for 48 h and then incubated with various concentrations of pratol (6.25, 12.5, 25, or 50 μM) and α-MSH (100 nM). After collection, the cells were lysed in RIPA buffer with protease inhibitor cocktail (1.0%). The cell lysates were vortexed every 10 min for 1 h and then centrifuged at 13,000× *g* for 10 min. The supernatants were collected and mixed with 2× Laemmli sample buffer (1:1) in an e-tube to prepare the samples for western blot analysis. Each sample was adjusted to an equal protein concentration (20 μg). Next, 20 μL of each sample per lane was loaded on sodium dodecyl sulfate-polyacrylamide gels. Separated proteins were transferred onto a polyvinylidene difluoride membrane, which was blocked with 5% non-fat skim milk in Tris-buffered saline containing 0.4% Tween 20 (TBST). The membranes were incubated with diluted primary antibodies (1:1000) for 24 h and then washed with TBST. They were then incubated with diluted secondary antibodies (1:3000) for 1 h. Protein bands were detected using an ECL kit.

### 4.7. Statistical Analysis

The results of the experiments were analyzed using student’s *t*-test. *p*-values < 0.05 (*) or 0.01 (**) were considered statistically significant. All data are expressed as percentages compared to the respective values of the control. Each result has been expressed as mean ± SD of at least three independent experiments.

## Figures and Tables

**Table 1:** Effects of some natural compounds on melanin production and the viability of B16F10 melanoma cells.

Compound	Concentration (μM)	Melanin Content (%)	Cell Viability (%)
7-Methoxycoumarin	12.5	108.5	99.7
	25	100.7	89.4
	50	96.5	93.2
	100	87.6	95.2
Bergamottin	12.5	101.2	100.9
	25	97.3	104.4
	50	79.2	110.9
	100	85.5	68.8
Synephrine	12.5	102.7	102.0
	25	100.2	96.2
	50	96.8	98.3
	100	116.1	98.1
Stigmasterol	12.5	100.0	99.1
	25	97.0	96.4
	50	105.0	98.2
	100	117.3	101.3
8-Methoxycoumarin	12.5	103.7	103.4
	25	112.1	105.5
	50	115.7	99.3
	100	130.3	98.1
Sciadopitysin	12.5	127.2	99.9
	25	130.6	99.8
	50	150.4	97.3
	100	153.7	94.5
Pratol	6.25	99.2	101.0
	12.5	108.9	99.2
	25	176.2	92.1
	50	208.7	88.4
